# FERMT1 suppression induces anti-tumor effects and reduces stemness in glioma cancer cells

**DOI:** 10.1007/s00432-024-05859-3

**Published:** 2024-07-08

**Authors:** Zhigang Pan, Chuhan Ke, Hanlin Zheng, Xiumei Guo, Wen Gao, Xinyue Huang, Chunhui Chen, Yu Xiong, Shuni Zheng, Feng Zheng, Weipeng Hu

**Affiliations:** Department of Neurosurgery, The Second Affiliated Hospital, Fujian Medical University, 34# zhongshan North Road, Quanzhou, Fujian 362000 China

**Keywords:** FERMT1, Glycolysis, Mitochondrial respiration, Cancer stem cells, WNT signaling pathways, Glioma

## Abstract

**Objective:**

Glioma is a leading cause of mortality worldwide, its recurrence poses a major challenge in achieving effective treatment outcomes. Cancer stem cells (CSCs) have emerged as key contributors to tumor relapse and chemotherapy resistance, making them attractive targets for glioma cancer therapy. This study investigated the potential of FERMT1 as a prognostic biomarker and its role in regulating stemness through cell cycle in glioma.

**Methods:**

Using data from TCGA-GBM, GSE4290, GSE50161 and GSE147352 for analysis of FERMT1 expression in glioma tissues. Then, the effects of FERMT1 knockdown on cell cycle, proliferation, sphere formation ability, invasion and migration were investigated. The influences of FERMT1 on expression of glycolysis-related proteins and levels of ATP, glucose, lactate and G6PDH were also explored. Furthermore, the effects of FERMT1 knockdown on cellular metabolism were evidenced.

**Results:**

Significant upregulation of FERMT1 in glioma tissues was observed. Silencing FERMT1 not only affected the cell cycle but also led to a notable reduction in proliferation, invasion and migration. The expression of glycolysis-associated proteins including GLUT1, GLUT3, GLUT4, and SCO2 were reduced by FERMT1 knockdown, resulted in increased ATP and glucose as well as decreased lactic acid and G6PDH levels. FERMT1 knockdown also inhibited cellular metabolism. Moreover, FERMT1 knockdown significantly reduced sphere diameter, along with inhibiting the expression of transcription factors associated with stemness in glioma cells.

**Conclusion:**

These findings demonstrated that FERMT1 could be an ideal target for the advancement of innovative strategies against glioma treatment via modulating cellular process involved in stemness regulation and metabolism.

**Supplementary Information:**

The online version contains supplementary material available at 10.1007/s00432-024-05859-3.

## Introduction

Gliomas are a highly aggressive and heterogeneous group of brain tumors that originate from glial cells within the central nervous system (CNS) (Ostrom et al. 2018; Tan et al. 2020). Despite the notable advancements in surgery, radiotherapy and chemotherapy, the prognosis of individuals with glioma remains discouraging, primarily attributed to the high incidence of tumor recurrence and the development of resistance to conventional therapies (Lim et al. 2018; Mahmoudi et al. 2019; Kamran et al. 2018). Therefore, there is a critical and urgent requirement to identify key molecular players involved in glioma progression and reoccurrence and unravel their underlying mechanisms.

Tumor progression and stemness have been identified as crucial factors contributing to the aggressive behavior and therapeutic resistance of glioma (Ma et al. 2018; Reya et al. 2001; Visvader and Lindeman 2008). Cancer stem cells (CSCs), a small subpopulation of cells within tumors, possess self-renewal and differentiation capabilities, which confers resistance to conventional therapies and contributes to tumor growth and recurrence in multiple types of cancers (Ma et al. 2018; Visvader and Lindeman 2008; Vlashi and Pajonk 2015). Moreover, the intricate relationship between cancer stem cells (CSCs) and the tumor microenvironment in glioma has been investigated (Boyd et al. 2021), and targeting CSCs in glioma is reported to benefit the efficacy of immunotherapy (Xun et al. 2021). Transcription factors and structural proteins were employed as markers of glioma CSCs, including SOX2 (Hemmati et al. 2003), OCT4 (Kaufhold et al. 2016), NANOG (Suvà et al. 2014), and MYC (Kim et al. 2010). Moreover, cell surface markers, including CD133 (Hemmati et al. 2003) and CD44 (Hassn Mesrati et al. 2021), were identified as the symbols of CSCs. Understanding the role of CSCs in regulating tumor progression and stemness is vital for developing targeted therapies to improve clinical outcomes.

FERMT1, also known as kindlin-1, is a member of the kindlin family of focal adhesion proteins (Siegel et al. 2003). FERMT1 assumes a pivotal role in mediating integrin-dependent cell adhesion and signaling, which encompasses a wide array of physiological and pathological processes including cancer progression. Its involvement in diverse cellular functions underscores the significance of FERMT1 in cancer development and progression (Li et al. 2022; Liu et al. 2017; Malinin et al. 2010; Has et al. 2009; Zhan and Zhang 2018). However, the involvement of FERMT1 in glioma and its effect on tumor progression and stemness remain largely uninvestigated.

To explore the functional significance of FERMT1 in glioma progression and stemness, we hypothesized that silence of FERMT1 in glioma cancer cells would suppress tumor growth, invasion, and stemness properties. To validate our hypothesis, we employed a combination of bioinformatic analysis as well as in vitro assays, including correlation between expression level and prognosis in patients, cell proliferation, migration, invasion, metabolism, and stemness assays, to evaluate the effects of FERMT1 on glioma progression. Our study contributed to the understanding of the mechanisms underlying tumor progression and stemness in this devastating disease. Furthermore, the identification of FERMT1 as a potential therapeutic target may open up new avenues for the development of novel and effective treatment strategies against glioma.

## Materials and methods

### Data collection

RNA-seq transcriptome data of patients with glioma in TCGA database (TCGA-GBM) was obtained from UCSC Xena (http://xena.ucsc.edu/). FERMT1 expression in GSE4290 (Sun et al. 2006), GSE50161 (Griesinger et al. 1950) and GSE147352 (Huang et al. 2021) datasets were obtained from GEO (https://www.ncbi.nlm.nih.gov/gds).

### Gene set enrichment analysis (GSEA)

GSEA was applied for enrichment of KEGG and Wiki pathways in which FERMT1 was involved. The analysis was performed using GSEA software (version 4.2.1) (https://www.gsea-msigdb.org/gsea/index.jsp) (Reimand et al. 2019).

### Cell Culture

To show the generality of the results of this experiment, we selected two glioma cell lines including U-251 MG (Cat. No. IM-H328) and T98G (Cat. No. IM-H215-1), these two cell lines and HEK293T (Cat. No. IML-092-1) cell line were acquired from Xiamen Immocell Biotechnology Co. Ltd. and maintained individually in corresponding media. U-251 MG and HEK293T cells were maintained in DMEM medium (Immocell, Xiamen, China), while T98G cells was cultured in MEM medium (Immocell, Xiamen, China). All media were supplemented with 10% fetal bovine serum (FBS, Gibco, South America) and 1% penicillin-streptomycin solution (Solarbio, Shanghai, China). Cell cultures were maintained in a CO_2_ incubator (Thermo Scientific TM) at 37 °C with a 5% CO_2_ atmosphere. Detection reports for the mycoplasma of the cells used were shown in Figure S1.

### Construction of stable FERMT1-knockdown cell lines

To silence FERMT1 expression, specific shRNAs targeting FERMT1 or a non-targeting control were inserted into pLKO.1-puro vector. The obtained plasmids were named shFERMT1 and shNC, respectively. The primers are as follows: shFERMT1-1 forward primer: 5´- CCGGGAAACAAGTGCTAAGTGTACCCTCGAGGGTACACTTAGCACTTGTTTCTTTTT-3´, shFERMT1-1 reverse primer: 5´- AATTAAAAAGAAACAAGTGCTAAGTGTACCCTCGAGGGTACACTTAGCACTTGTTTC-3´; shFERMT1-2 forward primer: 5´- CCGGGAGCAGCTGCTCTTACGATTTCTCGAGAAATCGTAAGAGCAGCTGCTCTTTTT-3´, shFERMT1-2 reverse primer: 5´-AATTAAAAAGAGCAGCTGCTCTTACGATTTCTCGAGAAATCGTAAGAGCAGCTGCTC-3´; shFERMT1-3 forward primer: 5´-CCGGCAGCTCTACAGTACCACATTACTCGAGTAATGTGGTACTGTAGAGCTGTTTTT-3´, shFERMT1-3 reverse primer: 5´-AATTAAAAACAGCTCTACAGTACCACATTACTCGAGTAATGTGGTACTGTAGAGCTG-3´.

Transfection was performed using Lipo-fectamine™ 2000 transfection reagent (Invitrogen, Cat. No. 11668500, Shanghai, China) according to the manufacturer’s instructions. Lentiviruses were generated through the transfection of HEK293T cells in 10-cm cell culture dish with 9 µg of shFERMT1 or shNC, 3 µg of pMD2G and 6 µg of pspax2 for 48 h. The lentivirus was enriched and the titer was determined as described previously (Li et al. 2015). In the presence of 8 µg/ml polypropylene, U-251 MG and T98G cells were infected with lentiviruses at a multiplicity of infection of 10. After 48 h, the medium was replaced with fresh medium, and puromycin was added at a final concentration of 0.5 µg/ml. After 96 h, cells were collected for FERMT1 expression analysis.

### Quantitative PCR analysis

TRIzol reagent (Invitrogen, Shanghai, China) was used to extract total RNA from cells according to manufacturer’s instructions. RNA reverse transcription kit (Cat. No. R122-01, Vazyme, Nanjing, China) was used to reverse transcribe RNA into cDNA. Subsequently, quantitative PCR was performed using the StepOnePlus Real-Time PCR system (Applied Biosystems, USA) and ChamQ SYBR qPCR Master Mix (Cat. No. Q311-02, Vazyme, Nanjing, China) with specific primers for the target genes, and the RNA content was analyzed using the 2^−∆∆CT^ method. The primer sequences are provided in Table 1.

**Table 1:** Primers for quantitative PCR

Primers	Sequence (5’-3’)
FERMT1 forward primer	ATATGATGCTGTCCGAATA
FERMT1 reverse primer	GCTAATGTGGTACTGTAGA
GLUT1 forward primer	TTGCAGGCTTCTCCAACTGGAC
GLUT1 reverse primer	CAGAACCAGGAGCACAGTGAAG
GLUT4 forward primer	CCATCCTGATGACTGTGGCTCT
GLUT4 reverse primer	GCCACGATGAACCAAGGAATGG
GLUT3 forward primer	TGCCTTTGGCACTCTCAACCAG
GLUT3 reverse primer	GCCATAGCTCTTCAGACCCAAG
18 S rRNA forward primer	AGGCGCGCAAATTACCCAATCC
18 S rRNA reverse primer	GCCCTCCAATTGTTCCTCGTTAAG
SCO2 forward primer	GACCACTCCATTGCCATCTACC
SCO2 reverse primer	CTCAAGACAGGACACTGCGGAA

### Western blotting

Cell lysis was performed using RIPA buffer (Beyotime Biotechnology, China) supplemented with a phosphatase inhibitor, and the cells were incubated on ice for 30 min. Subsequently, the samples were sonicated using a Bioruptor for 10 min. Protein extraction was followed by quantification of the protein concentration using a BCA kit (Cat. No. PA115-02, TIANGEN Biotechnology, Beijing, China). Next, the protein samples were denatured by heating at 95 °C for 10 min and loaded onto 4-16% SDS-PAGE gels at a concentration of 20 µg protein per well. Electrophoresis was conducted for 1.5 h at 90 V, and subsequently, electrophoretic transfer was performed for 1.5 h at 300 mA. PVDF membranes were used and blocked with 5% non-fat skim milk for 1 h at 25 °C. The membranes were then incubated overnight at 4 °C with primary antibodies (Table 2). Then, the membranes were incubated for 1 h at 25 °C with secondary antibodies (Table 2). After three washes, protein bands were visualized using an enhanced chemiluminescence reagent (Biorad, Shanghai, China) and captured using a Bio-Rad ChemicDoc machine.

**Table 2:** Antibodies for western blotting

Classification	Antibodies	Provider	Catalog number	Dilution
primary antibodies	Actin	Proteintech, Wuhan, China	20536-1-AP	1:5000
	FERMT1	Proteintech, Wuhan, China	22215-1-AP	1:3000
	GLUT1	Proteintech, Wuhan, China	21829-1-AP	1:3000
	GLUT3	Proteintech, Wuhan, China	20403-1-AP	1:3000
	GLUT4	Proteintech, Wuhan, China	66846-1-Ig	1:4000
	SCO2	Proteintech, Wuhan, China	21223-1-AP	1:4000
	MYC	Immunoway, Plano, American	YT0991	1:4000
	OCT4	Proteintech, Wuhan, China	11263-1-AP	1:3000
	NANOG	Proteintech, Wuhan, China	14295-1-AP	1:3000
	Bax	Proteintech, Wuhan, China	50599-2-Ig	1:3000
	Bcl2	Proteintech, Wuhan, China	26593-1-AP	1:2000
	Cleaved caspase 3	Proteintech, Wuhan, China	25128-1-AP	1:2000
	Caspase 3	Proteintech, Wuhan, China	19677-1-AP	1:2000
second antibody	HRP-conjugated goat anti-mouse IgG	Proteintech, Wuhan, China	SA00001-1	1:10000
	HRP-conjugated goat anti-rabbit IgG	Proteintech, Wuhan, China	SA00001-2	1:10000
HRP: horse radish peroxidase				

### Methyl thiazolyl tetrazolium (MTT) assay

Cell proliferation of U-251 MG and T98G cells were detected by MTT assay. After transfection with the shNC or shFERMT1 plasmid for 48 h, 6,000 cells were seeded in a 96-well plate per well. A final concentration of 0.5 mg/mL MTT reagent (Sigma, Shanghai, China) was added and incubated at 37 °C for 4 h. Subsequently, the medium was discarded, and dimethyl sulfoxide (Sigma, Shanghai, China) was added. The optical density (OD) value of was measured at a wavelength of 490 nm after a 4-hour incubation.

### 7-amino-actinomycin D (7-AAD) assay

U-251 MG or T98G cells were detached using trypsin and subsequently fixed in pre-chilled 95% ethanol at -20 °C for 12 h. Afterward, the cells incubated with 20 mg/mL 7-AAD (Solarbio, Shanghai, China) in PBS at 37 °C for 10 min. Following the incubation period, the cell apoptosis was analyzed using flow cytometry. Flow cytometry results were analyzed using Flowjo (BD Bioscience) to gate and calculate the proportion of cells in G_0_/G_1_, S and G_2_/M phase. All experiments were performed in triplicate.

### Sphere formation assay

For sphere formation assays, 2,000 cells per well were added in ultralow attachment plates. U-251 MG and T98G cells were cultured for a period of ten days in DMEM medium supplemented with 4 mg/mL insulin, 1:50 B27, 20 ng/mL EGF, and 20 ng/mL basic FGF. After 1.5 weeks, sphere counting was conducted under a microscope.

### Annexin V-FITC/PI assay

For apoptosis analysis, the Annexin V-FITC assay was conducted following the manufacturer’s instructions (Beyotime Biotechnology, Shanghai, China). U-251 MG and T98G cells were harvested by centrifugation at 200 × g for 5 min. Annexin V-FITC staining solution, PI staining solution, and binding buffer were added and gently mixed. After incubation for 15 min at 25 °C without light, apoptosis was assessed using flow cytometry (BD Sciences). Flow cytometry results were analyzed using Flowjo (BD Bioscience) to gate and calculate the proportion of Annexin V-FITC- /PI-, Annexin V-FITC+ /PI-, Annexin V-FITC- /PI + and Annexin V-FITC+ /PI + cells. All experiments were performed in triplicate.

### Colony formation assay

Cells were seeded in 6-well plates at a density of ~ 700 cells per well and cultured for 12 days. The cells were then fixed with 4% paraformaldehyde for 30 min and stained by 0.5% crystal violet for 15 min. The colonies were then washed and photographed. ImageJ (National Institutes of Health, Bethesda, MD, USA) was used for colony number calculation based on graph import and setting of the parameters. Biological triplicates were performed and analyzed for each group.

### Transwell assay

U-251 MG or T98G cells were transfected with FERMT1 and subsequently seeded in the upper transwell chambers (Corning Incorporated) at a density of 5 × 10^4^ cells per well, using serum-free medium. 100 µL of a 1:8 dilution of Matrigel (BD Sciences) was added to the upper chamber for invision detection, followed by incubation at 37 °C for 12 h. The lower compartment was filled with 600 µL of DMEM medium supplemented with 10% FBS. After incubation at 37 °C with 5% CO_2_ for 24 h, cells that had migrated to the lower chambers were fixed in 95% ethanol for 10 min, stained with 0.1% crystal violet solution for 10 min. The membranes were air-dried, photographed, and cell counts were performed using ImageJ software. For migration detection, Matrigel was not added, and the rest of the procedures were consistent with invasion assays. Three independent experiments were conducted for each experimental condition.

### Detection of glucose, lactate acid, G6DPH enzyme activity and pH

The content of glucose in supernatant was detected by blood glucose meter (Roche). Lactate acid level in supernatant was determined using kit from Nanjing Jiancheng Bioengineering Institute (Catalog number: A019-2-1) according to manufacturer’s protocol. Briefly, cell supernatant and reagents were added by order, and the OD values were determined using a microplate reader. G6DPH enzyme activity in cell lysates were performed using G6PDH activity assay kit (Cat. No. BC0260) from Solarbio life sciences according to manufacturer’s instructions. pH level in supernatant was detected using PHS-430 high precision intelligent pH meter (YIMA).

### Seahorse experiment

The measurement of oxygen consumption rate (OCR) and extracellular acidification rate (ECAR) was conducted following the procedures outlined in the published paper (Hu et al. 2023). Briefly, U-251 MG and T98G cells (40,000 cells per well) were seeded in XF96-well plates. The XF96 sensor cartridge was hydrated with calibration buffer and incubated at 37 °C overnight. After 12 h, the media were switched to serum-free DMEM (pH 7.4) containing 25.00 mM D-glucose and 4.00 mM glutamine. To measure OCR, oligomycin (1 µM final concentration), carbonyl cyanide 4-(trifluoromethoxy) phenylhydrazone (FCCP, 0.5 µM final concentration) and rotenone and antimycin A (1 µM final concentration) were administrated. For ECAR measurement, glucose (10 mM final concentration), oligomycin (1 µM final concentration), and 2-deoxy glucose (50 mM final concentration) were administrated. Subsequently, the measurement was performed using the Agilent Seahorse XFe96 system (Seahorse Bioscience, Billerica, MA). OCR and ECAR values were normalized to cell numbers for comparison between different groups.

### Flow cytometry

Cells were harvested and washed by PBS with 2% serum. Diluted FITC-conjugated anti-CD44 antibody (ab27285, Abcam) were applied to resuspend and incubate the cells for 30 min on ice. The cells were then washed, resuspended and analyzed by flow cytometry machinery to assess CD44 expression. Flow cytometry results were analyzed using Flowjo (BD Bioscience). Mouse monoclonal isotope antibody (ab91356, Abcam) was applied as control.

### Statistical analysis

For bioinformatics analysis, we utilized R software version 4.0.5 to conduct statistical analyses. To determine the significant expression of FERMT1 between normal and glioma samples, we employed the Wilcoxon signed-rank test. Additionally, the Kolmogorov-Smirnov test was used to assess the correlation between overall survival probability and FERMT1. GraphPad Prism 8.0 was employed to generate graphs and perform statistical analyses. For difference comparison analysis between two groups of parametric data, we applied the unpaired Student’s *t*-test. To quantify the results of Western blotting, we captured and analyzed photographs using ImageJ (Schneider et al. 2012). All values are expressed as the mean ± standard deviation (SD). * *P* < 0.05, ** *P* < 0.01, *** *P* < 0.001, and ****P < 0.0001.

## Results

### FERMT1 is a prognostic biomarker for glioma cancer and might be involved in stemness through cell cycle and WNT signaling pathways

**Fig. 1:**
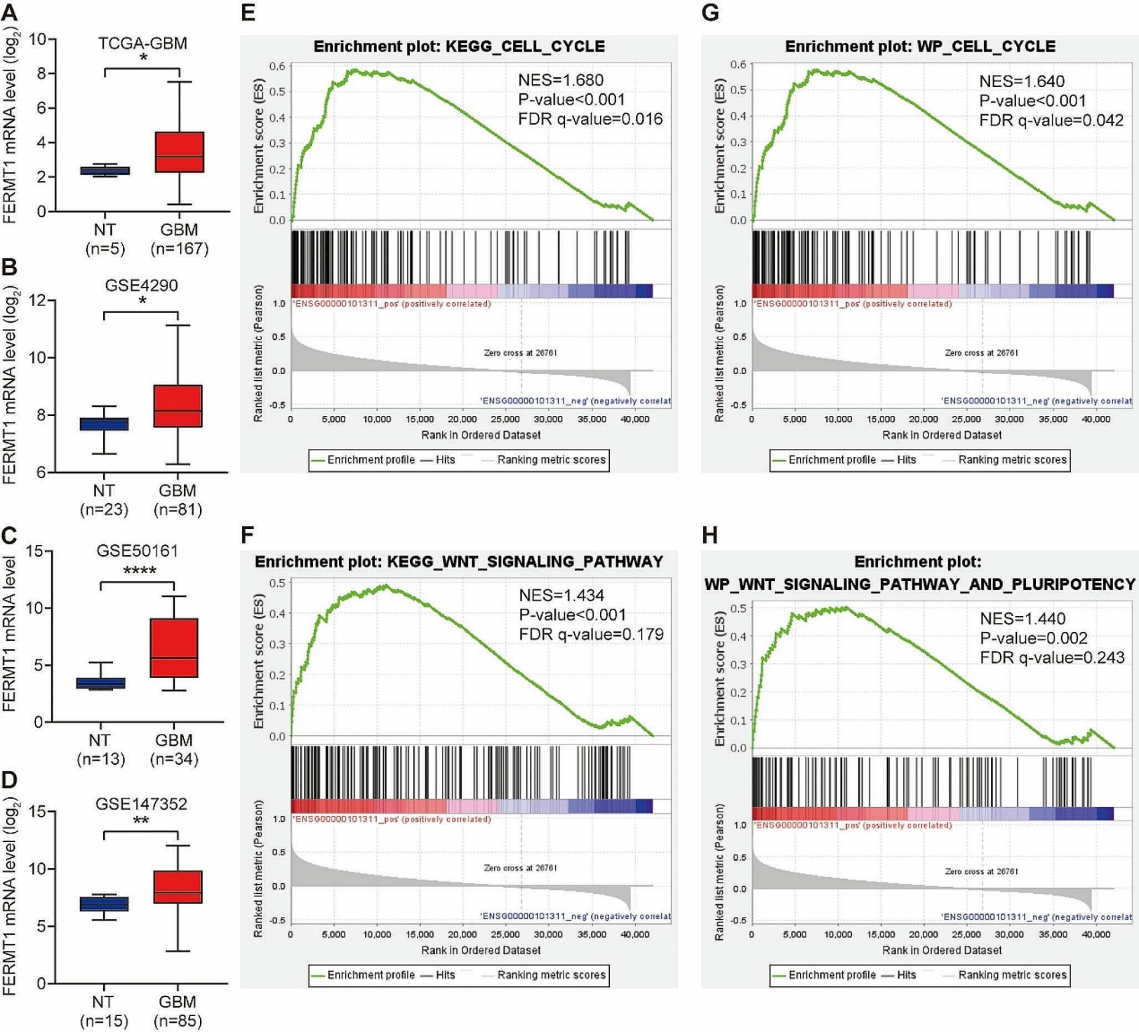
FERMT1 is a Prognostic Biomarker for Glioma Cancer and Regulates Stemness through Cell Cycle and WNT Signaling Pathways. A-D. The mRNA levels of *FERMT1* in glioma cancer cells from TCGA-GBM **(A)**, GSE4290 **(B)**, GSE50161 **(C)** and GSE147352 database **(D)**. **(E-H)** Gene set enrichment analysis (GSEA) results of enriched KEGG and Wiki pathways between high and low expression of FERMT1

To investigate *FERMT1* expression in glioma cancer tissues, we conducted a comprehensive analysis of mRNA expression data obtained from various datasets, including the TCGA-GBM, GSE4290, GSE50161 and GSE147352. From these transcriptome data, we demonstrated significant overexpression of *FERMT1* mRNA in glioma cancer cells (Fig. 1A and D). Through The Human Protein Atlas website (https://www.proteinatlas.org/), FERMT1 expression in the tissue of gliomas was queried, the results were shown in Figure S2, compared with the glial cells, FERMT1 was highly expressed in glioma tissues. To further explore the potential function of *FERMT1* in glioma, we analyzed the KEGG and WP pathways related to difference in *FERMT1* expression level. High expression of FERMT1 was annotated to be closely related to cell cycle and WNT signaling pathway (Fig. 1E and H). Overall, these results suggested that *FERMT1* exhibits high expression. Additionally, it may involve in CSC regulation since cell cycle and WNT signaling pathway was reported to be associated with stemness (Katoh and Katoh 2022). UALCAN database (https://ualcan.path.uab.edu/index.html) was used to explore the correlation between FERMT1 and survival time of glioma patients, the results were shown in Figure S3, with a cut off value of 50% and P value = 0.058, there was no significant difference in survival time of patients between high and low FERMT1 expression, the expression level of FERMT1 was not related to the survival time of patients.

### Inhibition of FERMT1 suppresses the malignant phenotypes of glioma cancer cells

**Fig. 2:**
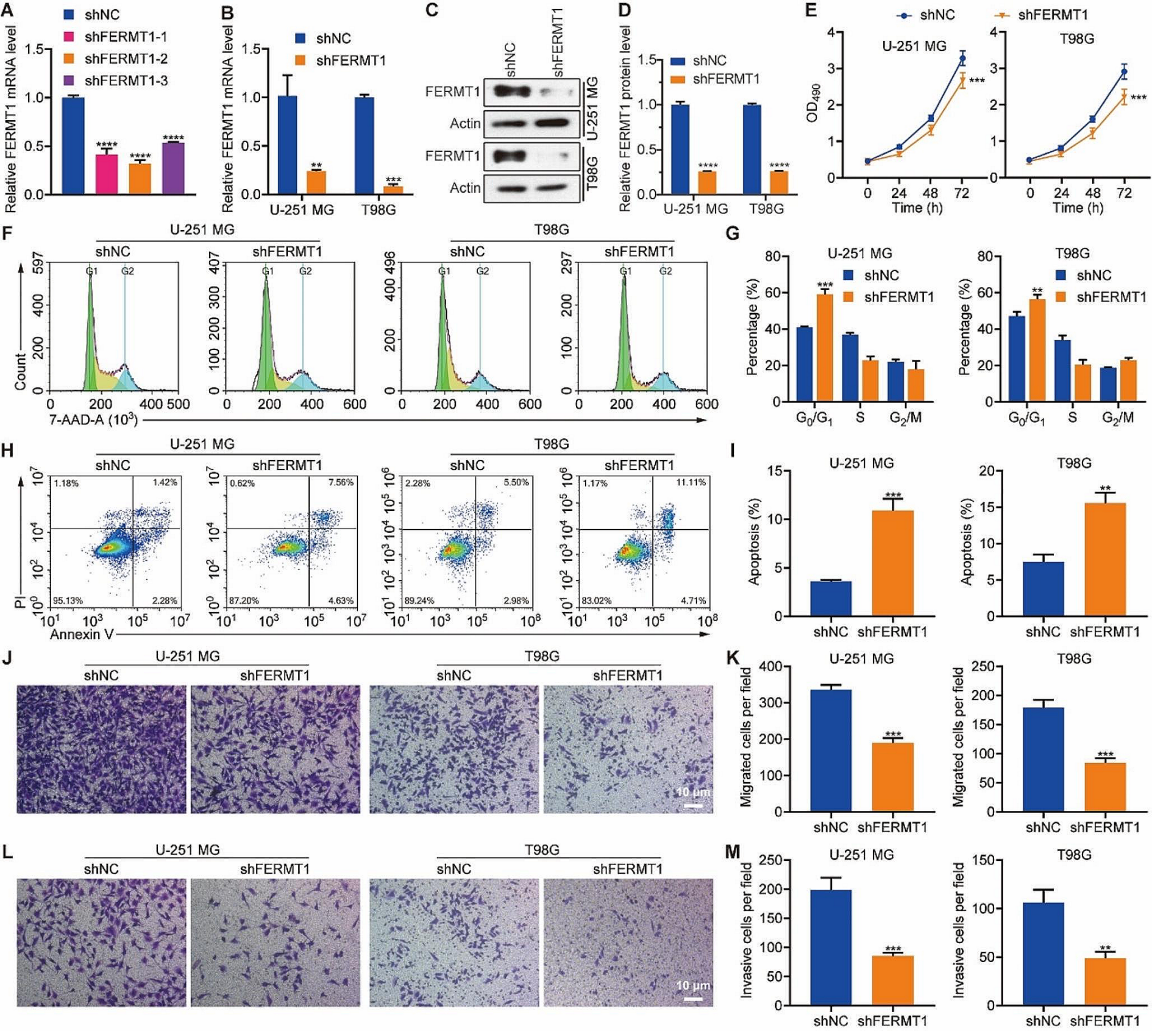
Inhibition of FERMT1 suppresses the progression of glioma cancer cells. **(A)** The mRNA level of *FERMT1* in 293T cells transfected with different shRNAs against FERMT1. **(B)** Quantification of *FERMT1* mRNA level in U-251 MG and T98G cells transfected with shRNA against FERMT1 or scramble control. **(C-D)** Representative images and quantification results of the protein level of FERMT1 in U-251 MG and T98G transfected with shRNA against FERMT1 or scramble control. **(E)** Cell proliferation results of U-251 MG and T98G cells transfected with shRNA against FERMT1 or scramble control. **(F-G)** Cell cycle analysis was conducted by 7-AAD staining and flow cytometry in U-251 MG and T98G cells transfected with shRNA against FERMT1 or scramble control. **(H-I)** Representative gating results and apoptosis quantification of Annexin-V-FITC/PI staining in U-251 MG and T98G cells transfected with shRNA against FERMT1 or scramble control. **(J-K)** Cell migration results in U-251 MG and T98G cells transfected with shRNA against FERMT1, or scramble control via transwell assay. **(L-M)** Cell invasion results in U-251 MG and T98G cells transfected with shRNA against FERMT1, or scramble control via transwell assay

To gain insights into the functional role of FERMT1 in glioma cancer cells, we employed shRNA to silence its expression and analyze the consequential phenotypes. We examined the knockdown efficiency of three shRNAs to silence FERMT1 in 293T cells, and most effective one was selected for transfection in U-251 MG and T98G glioma cancer cells (Fig. 2A). Both FERMT1 mRNA and protein levels were substantially reduced in cells transfected with the FERMT1-targeting shRNA compared to cells with non-targeting shRNA (shNC) (Fig. 2B and D). Cell proliferation analysis using MTT assay displayed significant decrease in the OD490 values of cells with FERMT1 knockdown compared to shNC cells, indicating an inhibitory effect on cell proliferation (Fig. 2E). Flow cytometry analysis via 7-AAD staining revealed that FERMT1 deficiency induced G_0_/G_1_ phase cell cycle arrest in both U-251 MG and T98G cells (Fig. 2F and G). Additionally, Cells with FERMT1 knockdown showed increased cell apoptosis rate, as indicated by Annexin-V/PI staining (Fig. 2H and I). Western blot was used to detect the protein expression of apoptotic proteins such as Bax, Bcl2, Cleaved Caspase 3, and Caspase 3 after FERMT1 knockdown in U-251 MG and T98G cells. As shown in Figure S4, in both cells, compared with shNC, Bax, Cleaved Caspase 3 expression was increased in shFERMT1, while Bcl2, Caspase 3 expression was decreased. Transwell assays also demonstrated that FERMT1 deficiency markedly reduced the percentages of migrated and invasive cells compared to shNC cells, which characterized the role of FERMT1 in promoting cell metastasis in glioma (Fig. 2J and M**)**.

### Knockdown of FERMT1 inhibits the mitochondrial respiration and glycolysis in U-251 MG and T98G cells

**Fig. 3:**
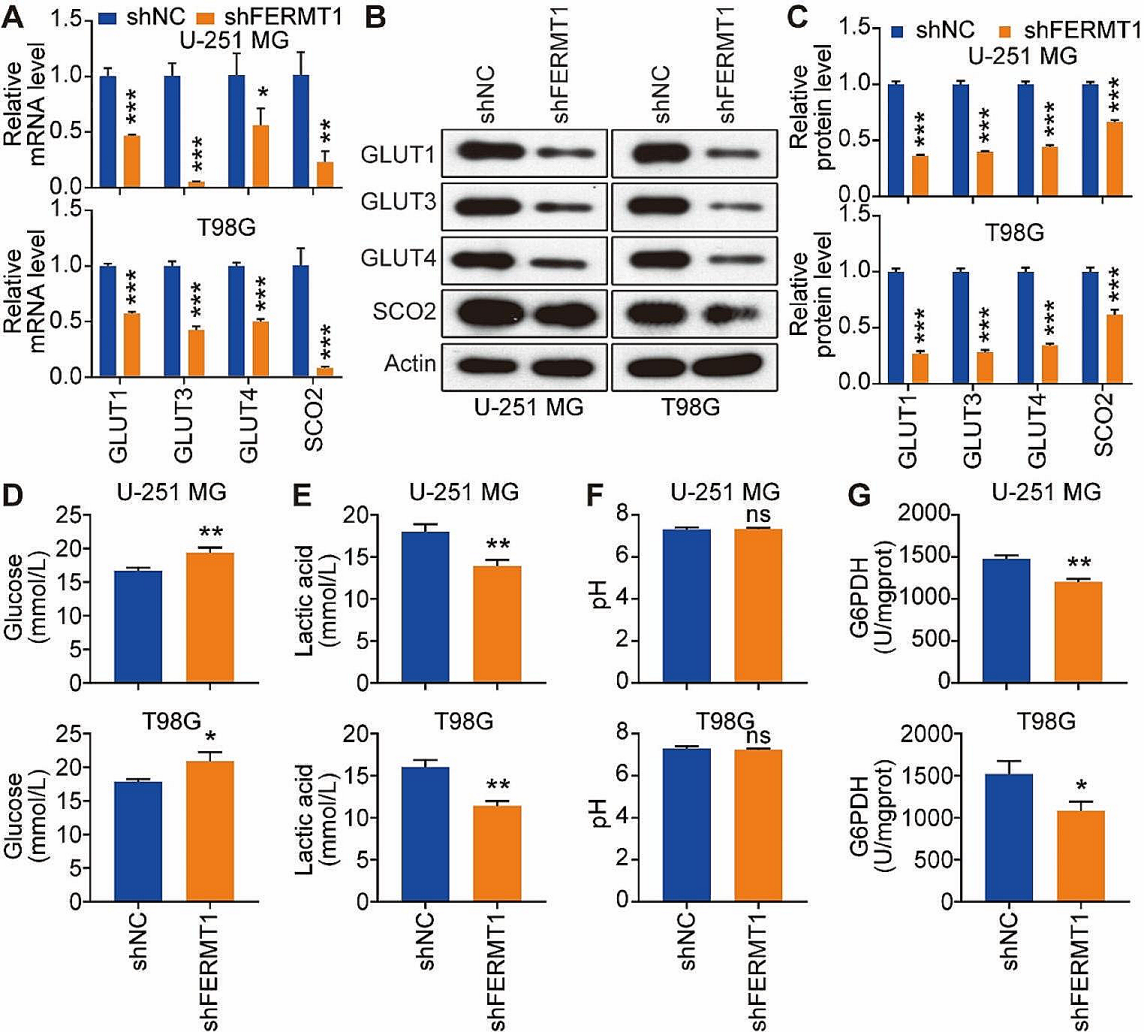
Knockdown of FERMT1 inhibit the glycolysis in U-251 MG and T98G cells. **(A-C)** mRNA and protein levels of GLUT1, GLUT3, GLUT4, and SCO2 in U-251 MG and T98G cells transfected with shRNA against FERMT1 or scramble control. **(D-F)** Glucose, lactic acid, and pH in the supernatant of U-251 MG and T98G cells transfected with shRNA against FERMT1 or scramble control. **(G)** G6DPH enzyme activity in the lysate of U-251 MG and T98G cells transfected with shRNA against FERMT1 or scramble control

Studies have provided evidence that WNT signaling influences cellular metabolic reprogramming, including the modulation of glycolysis and tricarboxylic acid (TCA) cycle activity (Leung and Lee 2022; Karner and Long 2017; Dong et al. 2022). We thus examined the expression levels of key transporters (GLUT1, GLUT3, GLUT4) and TCA cycle-related protein SCO2 in glioma cell lines. Surprisingly, we observed a substantial decrease in both mRNA and protein levels of GLUT1, GLUT3, GLUT4, and SCO2 upon FERMT1 knockdown in U-251 MG and T98G cells (Fig. 3A and C). To further validate the effect of FERMT1 knockdown on glycolysis, we examined the characteristics in cells and supernatant related to glycolysis. As shown in Fig. 3D and F, U-251 MG and T98G cells exhibited elevated levels of glucose and decreased lactate acid level in the supernatant, while the pH remained relatively unchanged following FERMT1 knockdown. We also found decreased G6PDH activity in cells with FERMT1 knockdown (Fig. 3G), which indicated inhibition of glucose metabolism. Together, these findings suggested that inhibiting FERMT1 might impede glycolysis and the TCA cycle, indicating the regulatory role for FERMT1 in these metabolic pathways.

**Fig. 4:**
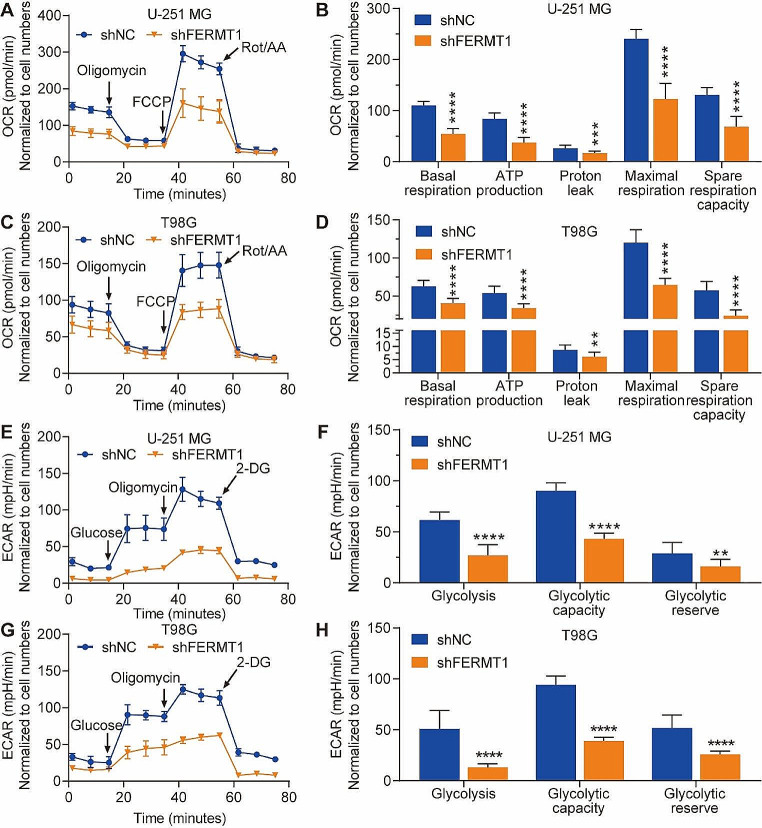
Knockdown of FERMT1 inhibit the mitochondrial respiration. **(A-D)** The oxygen consumption rate (OCR) under different conditions measured with addition of oligomycin, FCCP and mixture of rotenone and antimycin A, in U-251 MG and T98G cells with or without FERMT1 knockdown. **(E-H)** The extracellular acidification rate (ECAR) under different conditions measured with addition of o glucose, oligomycin and 2-DG, in U-251 MG and T98G cells with or without FERMT1 knockdown

To gain further insights into the effect of FERMT1 on cellular metabolism, oxygen consumption rate (OCR) and extracellular acidification rate (ECAR) were analyzed using a seahorse machine. Our results demonstrated that U-251 MG and T98G cells with FERMT1 knockdown exhibited significantly reduced OCR in terms of basal respiration, ATP production, proton leak, maximal respiration, and spare respiratory capacity, compared to control cells (Fig. 4A and D). Similarly, the ECAR, reflecting glycolysis, glycolytic capacity, and glycolytic reserve, was significantly downregulated following FERMT1 inhibition in U-251 MG and T98G cells (Fig. 4E and H). Collectively, these results demonstrated that knockdown of FERMT1 inhibited cellular metabolism in glioma cancer cells.

### FERMT1 regulates stem cell-like properties of human glioma cancer cells

**Fig. 5:**
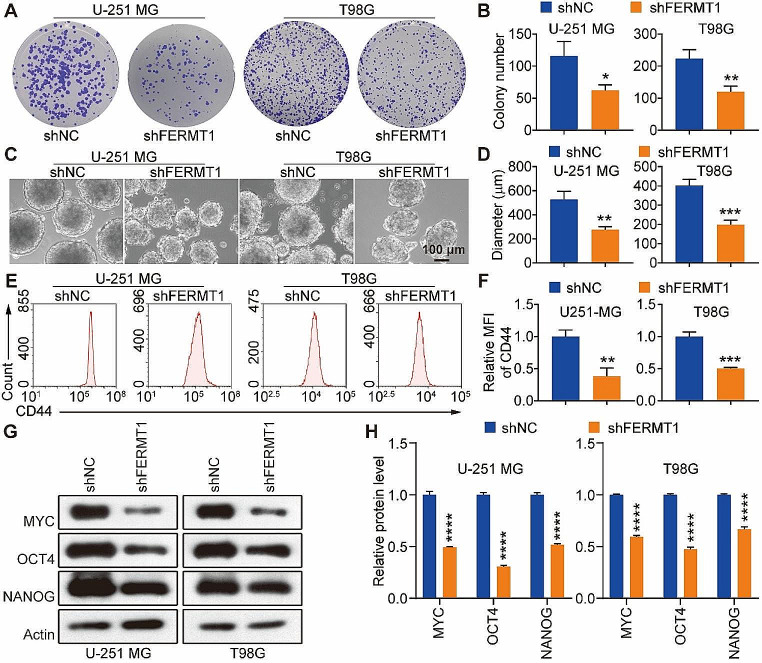
FERMT1 regulates stem cell-like properties of human in glioma cancer cells. **(A-B)** Representative images and quantification of clonogenicity of U-251 MG and T98G cells with or without FERMT1 deficiency. **(C-D)** Representative images and diameter measurement of the spheres formed by U-251 MG and T98G cells with or without FERMT1 deficiency. **(E-F)** Flow cytometry analysis of cell surface CD44 expression in U-251 MG and T98G cells with or without FERMT1 knockdown. **(G-H)** The effect of FERMT1 knockdown on the protein levels of stem cell markers, including MYC, OCT4 and NANOG in glioma cancer spheres

To investigate the involvement of FERMT1 in the maintenance of stem cell-like properties in human glioma cancer cells, we cultured U-251 MG and T98G cells with or without FERMT1 knockdown as 3D spheres. The colony formation assay demonstrated a significant inhibition in the formation and growth of colonies in both U-251 MG and T98G cells upon FERMT1 knockdown, indicating a crucial role of FERMT1 in promoting proliferation (Fig. 5A and B). Furthermore, to investigate the effect of FERMT1 knockdown on glioma CSCs, we examined the diameter of spheres formed by U-251 MG and T98G cells. FERMT1-deficient spheres displayed a significantly smaller size compared to controls, which suggested impaired sphere growth (Fig. 5C and D). To gain additional insights into the impact of FERMT1 knockdown on stemness-related characteristics, we performed flow cytometry analysis of stem cell markers. Remarkably, we observed significant reduction of CD44 in glioma cancer cells with FERMT1 knockdown, indicating that FERMT1 deficiency suppressed the formation of stem cell-like properties (Fig. 5E and F). Considering that pluripotent transcription factors, including MYC, OCT4 and NANOG, play critical roles in regulating the biological activities of CSCs, we analyzed the protein levels of these factors as well. FERMT1 knockdown significantly suppressed of MYC, OCT4 and NANOG expression in U-251 MG and T98G cells, which coordinately supported the regulatory role of FERMT1 in self-renewal and stem cell-like properties in glioma (Fig. 5G and H). In summary, our results collectively exhibited compelling evidence which supported the crucial involvement of FERMT1 in the regulation of stem cell-like characteristics in glioma cancer cells.

## Discussion

Gliomas constitute a substantial proportion of primary brain tumors, comprising approximately 30% of cases, and represent a major contributor to mortality accounting for about 80% of malignant brain tumors (Schwartzbaum et al. 2006). Survival outcomes in gliomas vary greatly depending on the grade. World Health Organization (WHO) grade 1 gliomas have the best relative survival, while CNS WHO grade 4 gliomas have the worst overall survival (OS) rate (Ostrom et al. 2019). Only 6.8% of patients diagnosed with WHO grade 4 gliomas survive for five years after diagnosis (Ostrom et al. 2019). Thus, identification of prognostic and predictive markers in glioma is of significant importance. It allows for the personalized treatment of patients based on their molecular or genetic characteristics, which may contribute to therapeutic strategies optimization and patient outcome improvement. Our study aimed to explore the potential role of FERMT1 as a prognostic biomarker for glioma. Several studies have implicated FERMT1 in colon cancer (Liu et al. 2017; Fan et al. 2011), nasopharyngeal carcinoma (Li et al. 2022), gastric cancer (Fan et al. 2020), oral squamous cell carcinoma (Wang and Chen 2021), and pancreatic cancer (Mahawithitwong et al. 2013). In the context of colon cancer, there is compelling evidence linking FERMT1 expression to tumor progression, metastasis, and an unfavorable prognosis (Liu et al. 2017; Fan et al. 2011). Similarly, FERMT1 overexpression has been associated with aggressive behavior and poor clinical outcomes in gastric cancer (Fan et al. 2020) and oral squamous cell carcinoma (Wang and Chen 2021). Through comprehensive analysis of multiple databases, we observed significant overexpression of FERMT1 in glioma cancer cells and upregulation of FERMT1 in tumor tissues, which is different with the FERMT1 expression in GSE108474 database (Lu et al. 2022). Our results demonstrated that FERMT1 high expression is associated with good clinical outcomes, suggesting its potential as a prognostic biomarker for glioma. These findings illustrated the involvement of FERMT1 in glioma development and progression, and the potential of FERMT1 to serve as a predictive marker for glioma prognosis. Identification of such markers helps clinicians in estimating disease progression, predicting survival rates, and making informed decisions regarding treatment intensity and follow-up protocols.

To validate the effect of FERMT1 in glioma, we employed shRNA-mediated knockdown to inhibit its expression in U-251 MG and T98G cells. Successful knockdown of FERMT1 was confirmed through significant reductions in FERMT1 mRNA and protein levels. Functional assays demonstrated that FERMT1 knockdown resulted in decreased cell proliferation, induction of G_0_/G_1_ phase cell cycle arrest, increased apoptosis and decreased migration/invasion capacity. These results are consistent with previous study of FERMT1 knockdown on HK1 which is a nasopharyngeal carcinoma cell line (Li et al. 2022). These findings strongly indicate that FERMT1 exhibits its effect on promoting malignant phenotypes of glioma cancer cells.

Moreover, we investigated the effect of FERMT1 knockdown on cellular metabolism, specifically glycolysis and mitochondrial respiration. We revealed that cells with FERMT1 knockdown exhibited reduced expression of glycolysis-related genes, indicating the inhibition of glycolysis. Furthermore, measurements of OCR and ECAR revealed impaired mitochondrial respiration and glycolytic function in FERMT1 knockdown cells. These findings firstly indicate that FERMT1 is involved in regulating cellular metabolism in glioma cancer cells, suggesting its potential as a therapeutic target for modulating metabolic activity in glioma.

Previous study illustrated that Kindlin-1, which encoded by FERMT1, controls Wnt and TGF-β availability to regulate cutaneous stem cell proliferation (Rognoni et al. 2014). We thus explored whether FERMT1 could regulate stem cell-like properties in glioma cancer cells. FERMT1 knockdown resulted in the inhibition of colony and sphere formation, reduced sphere size, and down-regulation of stem cell markers CD44, MYC, OCT4, and NANOG. These results indicate that FERMT1 contributes to maintaining stem cell-like properties in glioma cancer cells, which may contribute to tumor progression, therapeutic resistance and disease reoccurrence. The Wnt pathway has been reported to play a crucial role in the occurrence and development of a variety of diseases. A large number of studies have shown that the Wnt pathway plays an essential role in glioma. For example, in the study by Yuan Xie et al., Wnt pathway regulates MFSD2A-dependent drug delivery in glioma through endothelial transcellular action. Inhibition of Wnt signaling pathway can increase the sensitivity of tumors to drug therapy, so targeting Wnt pathway may provide promising opportunities for successful treatment of glioma (Xie et al. 2023). In addition, FERMT1 has been shown to play a cancer-promoting role by regulating the Wnt pathway. FERMT1 activates the Wnt/β-catenin signaling pathway by reducing the phosphorylation level of β-catenin, enhancing the nuclear translocation of β-catenin and increasing the transcriptional activity of β-catenin/TCF/LEF, thereby promoting the epithelial-mesenchymal transition (EMT) in colon cancer metastasis (Liu et al. 2017). At present, the molecular mechanism of FERMT1 affecting glioma through Wnt pathway has not been studied, and whether FERMT1 also affects downstream pathways by regulating β-catenin needs to be explored by further experiments.

The role of EMT in glioma cannot be ignored. In the study of Yang Nan et al., the results showed that miRNA-451 reduced EMT and metastasis of glioma cells in vitro and vivo by targeting CAB39 to inhibit PI3K/Akt/Snail signaling pathway, therefore, miR-451 may be a new target for glioma treatment (Nan et al. 2021). It has been reported that there is an interaction between EMT and cancer stem cell pathways, and the occurrence of EMT contributes to the generation of cancer stem cells (Fuxe et al. 2010). In the study by Xiangzhou Chen et al., tumor-associated macrophages promoted EMT occurrence in triple-negative breast cancer by activating CCL2/AKT/β-catenin signaling pathway and enhanced cancer stem cell characteristics, which provided a new strategy for tumor diagnosis and treatment (Chen et al. 2022). It has been reported that FERMT1 and EMT play a regulatory role in cancer, for example, FERMT1 inhibits the migration, invasion and EMT of oral squamous cell carcinoma cells by inhibiting the PI3K/AKT signaling pathway, which becomes a new target for the anti-metastasis of cancer (Wang and Chen 2021). Knockdown of FERMT1 significantly reduced the proliferation, migration and invasion of nasopharyngeal cancer cells by regulating the occurrence and development of EMT Therefore, elucidating the mechanism of how FERMT1 regulates EMT in glioma to affect stem cell properties and then play a role in tumors is worth exploring.

In conclusion, we highlight the potential of FERMT1 as a prognostic biomarker for glioma and its involvement in regulating tumor progression, cellular metabolism and stemness in cancer cells. Further investigations are still necessary to elucidate the underlying molecular mechanisms by which FERMT1 exerts its effects and to explore its feasibility as a therapeutic target in clinical settings.

### Electronic supplementary material

Below is the link to the electronic supplementary material.

Supplementary Material 1

## Data Availability

The datasets used or analyzed during this study are available from the corresponding author on reasonable request.
