# Supplementary material for: FERMT1 suppression induces anti-tumor effects and reduces stemness in glioma cancer cells
Source: J Cancer Res Clin Oncol. 2024 Jul 8;150(7):338. doi: 10.1007/s00432-024-05859-3 (PMC11231014; doi:10.1007/s00432-024-05859-3)
Supplement: Supplementary file 1 — Supplementary Material 1 [file 432_2024_5859_MOESM1_ESM.docx]

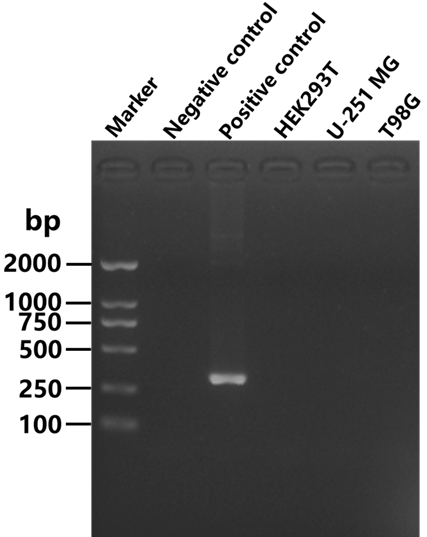


**Figure S1.** Report of mycoplasma detection in U-251 MG, T98G and HEK293T cell lines.


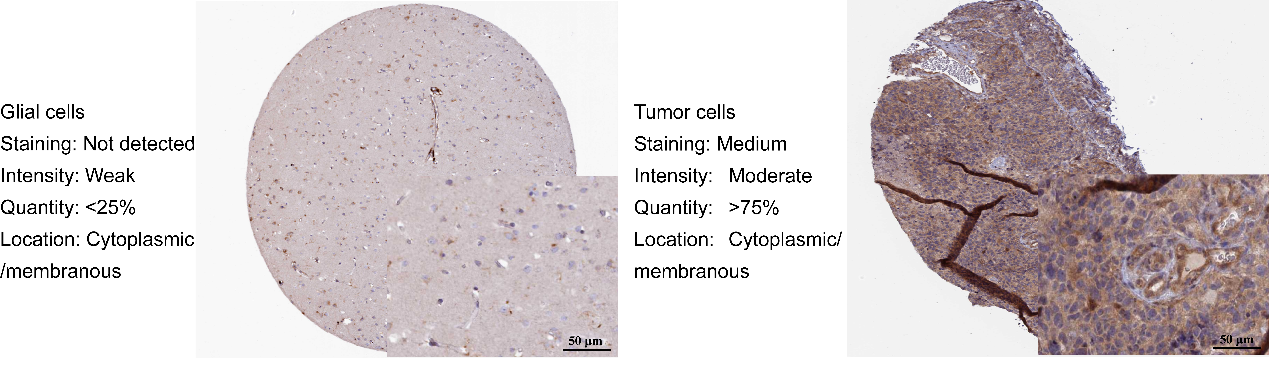


**Figure S2.** The expression of FERMT1 in glial cells and tumor cells was queried online using The Human Protein Atlas.


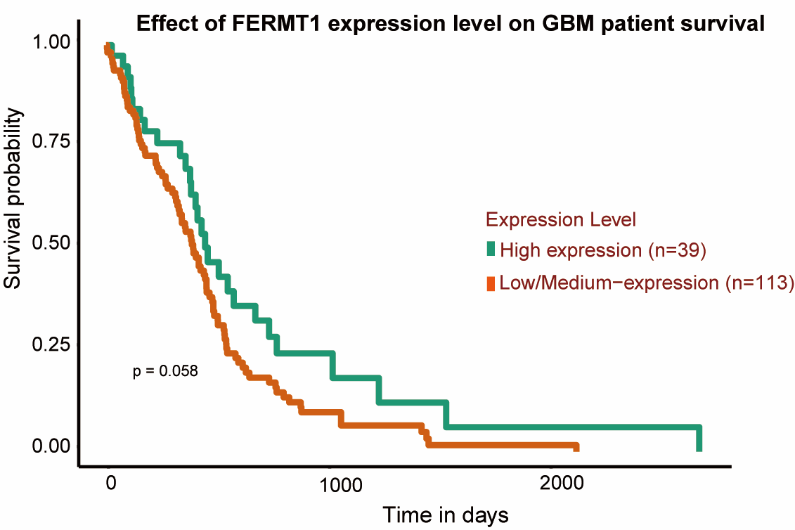


**Figure S3.** Online analysis of correlation between FERMT1 and survival time of glioma patients by using UALCAN databases.


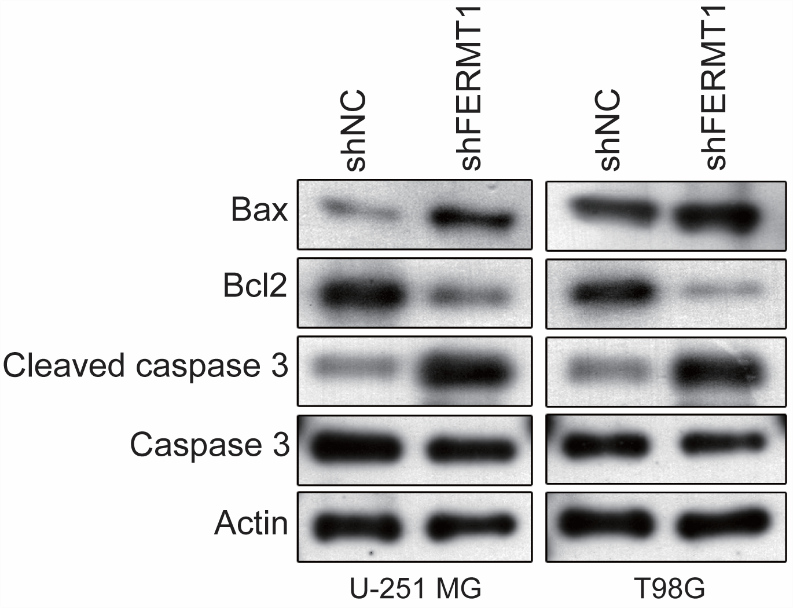


**Figure S4.** Protein levels of Bax, Bcl2, Cleaved caspase 3 and Caspase 3 in U-251 MG and T98G cells transfected with shRNA against FERMT1 or scramble control.
